# The Dictionary of Foods and Drinks

**Published:** 1855-06

**Authors:** 


### THE DICTIONARY OF FOODS AND DRINKS;

### A COMPENDIUM OF ALIMENTARY SUBSTANCES IN USE THROUGHOUT THE WORLD: THEIR HISTORIES, DIETETIC PROPERTIES, AND ADULTERATIONS.

### PART I.

ABANGA, the native name of a palm, sometimes called Thernel's restorative, but most commonly ady, or *palma ady*, and by the Portuguese *carvoces* or *cariosse*. The abanga is a tall plant of the endogenous class, natural order *Palmaceæ*. It grows in the West Indies, especially in the island of St. Thomas. Three articles of diet are obtained from this tree by the natives: 1. An intoxicating fermented liquor from the juice furnished by the thick branching head of the tree; 2. An oily substance, of a saffron colour, resembling butter, extracted from the kernels by immersion in hot water; 3. The kernels themselves, which are given to debilitated people in the proportion of from three to four a day, as a general restorative, as cod-liver oil is given in England. The products of this tree have never been used in this country as aliments.

ABELMOSCHUS, or Syrian manna, a slender herbaceous plant of the natural order *Malvaceæ* (Linnean class and order *Monadelphia Polyandria*). The seeds are aromatic, and are used by the Arabs to mix with coffee.

ABSINTHE, the common name of a liquor used in France, prepared from wormwood (*absinthium*) tops, sweet flag, aniseed, angelica root, and alcohol, and coloured green with the leaves or the juice of smallage, spinage, or nettles. The liquor is used as gin and bitters is employed in this country. Several adulterations enter into this liquor, which are very prejudicial to health. M. Derheims refers to sulphate of copper (blue vitriol) as having been used as the colouring material; and M. Stanislas Martin states that he has found chloride (or butter) of antimony used for the same purpose. This last adulteration is, however, doubtful. (*Chevallier.*)

ACAJOU, also acajaiba, a name for the cashew-nut. See *Cashew-nut.*

ACARUS (*a* not; *κείρω*, to cut; because it cannot, from its smallness, be divided), the technical name of an animal of the class *arachnida,* commonly known as the mite. The various species are widely distributed. Some infest the human subject (giving rise to the disease called *scabies*), as well as various animals. According to Cuvier, they may be found in water, flour, dried meat, and putrid animal matters; but the best known species is that which infests old dry cheese. In relation to foods, considerable interest has been added to these animals by the discovery, by Dr. Hassall, of the presence of a species of them in brown sugar. This species is termed, by the discoverer, the *acarus sacchari,* or sugar-mite. It is plainly visible to the unaided sight; and, when present in sugar, may be thus detected. Two or three teaspoonfuls of sugar should be dissolved in a wineglass of tepid water, and the solution allowed to remain at rest for an hour. At the end of the time, the acari will be found, some on the surface of the liquid, some adhering to the sides of the glass, and others at the bottom, mixed up with the dark sediment. According to Hassall, the acarus is commonly present in nearly all brown sugar as imported into this country, and in raw or Muscovado sugar; but not in lump-sugar, sugar-candy, or the white sugars of the East Indies, prepared by filtration. It owes its existence in sugar to the presence of vegetable albumen; without which it cannot exist. Its absence is an indication of the purity of the sugar. It is not generally present in treacle; the thick fluidity of this article preventing it from obtaining atmospheric air. As a question of adulteration, it is doubtful whether any of the acari are injurious to the body, when swallowed. It is stated on no less an authority than that of Cuvier, that they have been found in the brain and the eyes of man; but this is questionable, as they cannot exist without atmospheric air. An interesting point has, however, been raised by Dr. Hassall, whether the disease known as grocer's itch, and which particularly affects those who are engaged in the handling of sugar, may not arise from the direct introduction under the skin of the *acarus sacchari*. The *acarus farinæ*, or meal-mite, so named, and first accurately described by the same author, infests damaged flour. Its presence is rare; and when found, indicates that the flour is not fit for consumption. (*Hassall on Foods;* and *Lancet for* 1851, vol. i, pp. 79 and 387.)

ACETARIA, a technical term for ordinary salads.

ACETARIUM SCORBUTICUM, an old medicinal diet made from the leaves of a species of scurvy-grass (*cochlearia*), a salt procured from the same plant, sugar, and orange-juice. It was recommended to be employed by scorbutic patients, by dipping their food in it; and formerly had a great reputation.

ACID, (*aceo*, to be sour,) in common parlance, any sour substance. In chemistry, the term is applied to certain compound substances which are capable of forming salts with alkalies, alkaline earths, the oxides of metals, and organic bases. Acids may be derived from animal, vegetable, and mineral sources; and we therefore hear of animal acids, (as butyric); of vegetable acids, (as tartaric and gallic); and of mineral acids, (as sulphuric and manganic). As a general rule, acids consist chemically of an elementary substance, or a compound having elementary properties, combined with oxygen or hydrogen. Acids are also divided into *organic*, or those derived from the animal and vegetable kingdoms; and *inorganic*, or those derived from mineral matter. They may exist in three forms; in that of gas, carbonic acid, of liquids, (as common sulphuric acid, or oil of vitriol,) and of solids, (as tartaric acid). Many of the solid acids take crystalline shapes. They have the property of turning vegetable blues red. Their powers of affinity for bases vary in different acids; thus, tartaric acid, when mixed with a solution of an alkaline carbonate, as carbonate of soda, from its greater affinity for the base soda, displaces the carbonic acid, which escapes with effervescence while the tartaric acid seizes itself on the soda.

Indirectly, and sometimes directly, some of the vegetable acids are largely consumed in daily use. They exist in a state more or less free in most ordinary ripe fruits; and the tartaric, the citric, and the acetic are used in their separate forms; the latter for pickling and salads, and the two former for flavouring drinks, as in lemonade and sherbet. Soda water is nothing more than a solution of carbonate of soda, strongly impregnated by simple pressure with carbonic acid. An agreeable vegetable acid, called malic, exists in large quantities in cider, in common rhubarb, and in many fruits, from which it might possibly be obtained at a very moderate price. Some vegetable acids of a poisonous nature, as oxalic, also exist in various edible substances; but either combined with a base or in such small quantities when free as not to prove detrimental to life.

The physiological action of acids on the living body is not well understood. Some of the mineral acids, as the sulphuric, nitric, and hydrochloric, are used extensively in medicine, as tonics or restoratives. Sulphuric acid is used as an adulteration of vinegar, and of some of the alcoholic drinks, especially brandy and gin. In brandy, the acid is added to give to the liquor the odour characteristic of age, by hastening the formation of ether, which arises by a natural chemical change in true old brandy. Girardin and Morin found in thirty-five specimens of brandy collected from different sources in Rouen, twenty-one in which sulphuric acid was present. When existing free in spirituous drinks, it cannot fail to prove more or less injurious to those who imbibe them. The same acid is used in the adulteration of gin; and in the manufacture of British gin it is systematically employed, together with rectified spirit, oil of turpentine, oil of almonds, juniper berries, loaf sugar, and alum. In this process it enters into combination, and ceases to exist in the free state.

Some of the vegetable acids, as the acetic and tartaric, have long been used as medicinal and alimentary agents. To acetic acid has been attributed various important properties. It has been supposed to be a preventive of plague, cholera, and scorbutic diseases, and a cure for scarlet fever and other febrile diseases.

The presence of vegetable acids in food is necessary. In their absence, diseases of the class commonly known as scorbutic seem inevitable. Thus scurvy arises more commonly from the absence of fresh vegetable substances in which acids are present, than from any other cause. Drs. Aldridge and Garrod are, however, of a different opinion; and maintain that it is the potash contained in vegetables which prevents the scorbutic tendency, and not the acids. This theory is not as yet generally admitted.

The acid substances, in their bearings on food, are important as giving rise accidentally to the formation of poisons: since they enter into various injurious combinations with lead and copper, metals commonly used in the formation of water-pipes and of culinary vessels. Acetic, citric, tartaric, and malic acids, all act on lead or copper with more or less power, forming soluble salts, highly injurious to life. Hence the preservation or preparation of articles of food containing vegetables in leaden or copper vessels, or glazed earthenware (which contains large quantities of oxide of lead), is always liable to produce danger. Milk, rum, wine, cider, and broth and other fatty matters, which either contain acids originally, or generate them during decomposition, yield, in leaden or copper vessels, poisonous salts. Heller has shewn, however, that milk and ordinary vegetables may be boiled in copper vessels with impunity, and do not dissolve the metal unless allowed to cool in it, and stand for some time. In using these vessels, therefore, for this purpose, the liquid should be poured off at once, and the vessel kept scrupulously clean. The action of acids on pure tin is very feeble, and the products are in too small quantity to do harm, even if they were poisonous; but the ordinary tin of commerce is alloyed with arsenic, copper, and lead, and the acids form deleterious compounds with all of these. Pewter, being a mixture of tin and lead, is subject to the action of acids; and hence precautions should be used regarding it. No alimentary substance containing an acid should ever be kept or put in a copper or leaden vessel, which is coated with an oxide of either metal. Mitchell refers to the common practice of introducing a piece of copper, as a penny-piece, into the water in which vegetables are being boiled, for the purpose of communicating to them a bright green colour. The practice is as dangerous as it is absurd.

When it is suspected that articles of food containing acids have become contaminated with the poisonous salts, copper, lead, or other metals, various chemical tests will readily detect the adulteration. Lead is precipitated from the solutions of its salts by transmitting through the solution, previously acidulated slightly with nitric acid, a stream of sulphuretted hydrogen, which throws down a black precipitate: liquor ammoniæ, liquor potassæ, and dilute sulphuric acid, form a white precipitate. If iodide of potassium be added to the solution, a bright yellow compound iodide of lead, is formed. Red chromate of potash forms a similar precipitate. If pieces of iron or zinc are suspended in a solution of salts of lead, this metal will adhere to them in the form of crystalline plates—forming, if in large quantity, a beautiful arborescent appearance.

Copper forms, under the like circumstances, a black precipitate with hydrosulphuric acid; a deep blue colour with ammonia; a brown precipitate with ferrocyanide of potassium (yellow prussiate of potash); and it is thrown down in a metallic form when metallic iron or tin is placed in a solution of its salts.

ACID, CITRIC, a vegetable acid existing in many fruits and vegetables, especially lemons and limes, from which it is separated by adding chalk (carbonate of lime) to the juice. By this proceeding a citrate of lime is formed. This citrate of lime is then treated with sulphuric acid, which seizes on the lime, and leaves the citric acid in solution, whence it is obtained by evaporation. It crystallises in rhomboid prisms; its specific gravity is 1.617. It is soluble in alcohol, in three-fourths of its weight of cold water, and in half its weight of boiling water. It is used in dyeing, for removing rust spots (forming with the iron a soluble citrate of iron). It is used extensively in medicine, combined with alkalies; and has been much employed in the form of lemon juice, in the treatment of rheumatic fever. Lemon juice, mixed with water and sugar, forms lemonade; and the purified acid is employed for giving an acid flavour to sweetmeats. Citric acid sometimes contains sulphuric acid, salts of lead, and, according to M. Risler, copper. It is adulterated with oxalic acid, tartaric acid, and sulphate of lime; principally with tartaric acid. According to Chevallier, the fraudulent manufacturers dissolve together the citric and tartaric or oxalic acids, and thus produce a crystal, having sufficient resemblance to that of citric acid to be at first sight mistaken for it. These adulterations are detected by the following tests:— 1. The crystals of tartaric acid are longer and smaller than those of citric acid: those of oxalic acid appear foliated, and are not so solid nor transparent. 2. When the acids have been crystallised together, chloride of potassium, or the acetate, neutral tartrate, or carbonate of potash, will throw down from a solution a crystalline or pulverulent precipitate of cream of tartar or acid oxalate of potash. 3. M. Gaffard has shewn that tartaric acid may be detected by adding drop by drop some of the suspected solution to lime water: the citrate of lime is very soluble in water, while the tartrate is very imperfectly so. 4. Salts of lime are detected by neutralising the acid in solution with ammonia, and dividing the fluid into two portions: to one of which oxalate of ammonia is added, and to the other chloride of barium. If a precipitate is formed in both vessels, sulphate of lime is present: if only in that to which oxalate of lime has been added, citrate of lime is present, which has arisen in the manufacture of the acid. Sometimes mere saturation with ammonia is sufficient to precipitate the sulphate of lime, which is only held in solution by the citric acid. 5. Sulphuric acid is detected by adding to the solution acetate of lead; any precipitate formed which is insoluble in nitric acid is sulphate of lead. This acid is also detected by the addition of chloride of barium, and by the tendency of the impure citric acid to absorb water from the atmosphere. 6. Lead is detected by sulphuretted hydrogen, which gives a black precipitate; of iodide of potassium, which forms yellow iodide of lead; and of chromate of potash, which forms yellow chromate of lead. 7. Copper is detected by forming a blue colour with ammonia; a brown precipitate with yellow prussiate of potash; and by being thrown down in a metallic form when a piece of iron or tin is placed in a solution containing any of its salts.

ACID, TARTARIC. This acid exists, like the citric, in many fruits, but principally in the grape, from the juice of which it is mainly obtained. In this juice it exists, combined with potash, in the form of bitartrate: this salt, being insoluble in alcohol, is thrown down in the fermentation of wine in an impure state, and is known under the name of *argol*, which, when purified, is called *cream of tartar*. Tartaric acid is obtained by dissolving cream of tartar in boiling water, and adding chalk until effervescence ceases. Two compounds result: tartrate of lime, which is precipitated; and tartrate of potash, which remains in solution. The solution, being poured off, is treated with chloride of calcium (hydrochlorate of lime), which gives rise to the formation of a precipitate of tartrate of lime, which is removed and mixed with the former precipitate. To this precipitate, very dilute sulphuric acid is added, which seizes on the lime, and sets the tartaric acid free in solution. The acid is then crystallized by evaporation, and purified by further solution and recrystallization. Its crystal is an oblique rhombic prism. Its specific gravity is 1·75. It is soluble in alcohol, but more so in water: it dissolves in five times its weight of water at 60°, and in twice its weight of water at 212° Fahr. Tartaric acid is used for much the same purposes as the citric, but more extensively, in consequence of its greater cheapness. It enters into the formation of seidlitz powders, sherbet, &c. Dr. Hassall states that it is used, in combination with carbonate of soda, in making unfermented bread, under which circumstance the bread becomes impregnated with the purgative salt, tartrate of soda; the carbonic acid escaping and lightening the bread. He also states, with Dr. Pereira, that what are called *egg* or *baking powders* are composed of carbonate of soda, tartaric acid, and potato-flour, coloured with turmeric. Tartaric acid when badly prepared may contain sulphuric acid, sulphate of lime, tartrate of lime, lead, and copper. It is adulterated in commerce with cream of tartar, with an acid sulphate of potash, and with lime. These may be detected in the following manner. 1. Sulphuric acid is recognised by the addition of chloride of barium or acetate of lead, as in citric acid. 2. Sulphate and citrate of lime are left undissolved in an alcoholic solution of the acid. These salts, too, when dissolved in boiling water, give a white precipitate, with oxalate of ammonia and chloride of barium. 3. Lead is detected by the same tests as when it is present in citric acid. 4. Copper in solution with the acid is indicated by a blue colour on the addition of ammonia; and yellow prussiate of potash produces a brown colour or precipitate. 5. Cream of tartar is nearly insoluble in cold water; and if the acid containing this salt is burnt, carbonate of potash is formed, which effervesces with acids, and forms a yellow precipitate with chloride of platinum. 6. Acid sulphate of potash is insoluble in alcohol, and is not destructible by heat. It is readily detected by means of chloride of barium. 7. When lime is present, carbonate of lime is left by incineration; this effervesces with acids, is precipitated by oxalate of ammonia, and when exposed to a stronger heat, is changed into caustic lime.

ACID DROPS, a well known sweet meat, composed of a vegetable acid, with water, sugar, and essential oil. The acid drops sold in the shops are generally of a whitish opalescent appearance, have an agreeable acidulated flavour, and are, in some cases, very deliquescent. They only act on the body by virtue of the acid they contain, which is generally the tartaric.

It has been stated that the acidulated drops are sometimes adulterated with mineral acids and other deleterious ingredients. This statement, however, we believe to be incorrect, (at least, in regard to the acid drops used in this country.) The following is an account of the composition of ten samples of these drops, procured from different sources, and which were submitted to Mr. Rodgers for chemical analysis.

No. 1.—Procured from Gunter's, Motcomb Street. A very pure sample, consisting of pure tartaric acid, 3·75; sugar, water, and essential oil, 96·25.

No. 2.—Procured from Grange's, 176, Piccadilly. A very pure sample, consisting of pure tartaric acid, 1·4; sugar, water, and essential oil, 98·6.

No. 3.—Procured from Bainbridge's, 148, Strand, 1½*d.* per oz.—A very pure sample, consisting of pure tartaric acid, 3; sugar, water, and essential-oil, 97.

No. 4.—Procured from the same place, but only 1*d.* per oz. Also a pure sample, being, as stated, of the same quality, but with less acid. Analysis gave:—pure tartaric acid, 2·47; sugar, water, and essential oil, 97·53.

No. 5.—Procured from Meggeson & Co., 61, Cannon Street. A pure sample, consisting of pure tartaric acid, 2·3; sugar, water, and essential oil, 97·7.

No. 6.—Procured from Little's, 16, South Audley Street. A pure sample, consisting of pure tartaric acid, 1·5; sugar, water, and essential oil, 98·5.

No. 7.—Procured from Sumner's, Marylebone Lane. Not so pure as the preceding samples, the sugar used having been less carefully clarified. Analysis gave:—tartaric acid, 1·4; sugar, water, and essential oil, 98·6.

No. 8.—From Braitling's, 26, Coventry Street. Similar to the last, but with a larger amount of acid. Analysis gave:—tartaric acid, 2·1; sugar, water, and essential oil, 97·9.

No. 9.—From a small shop in Hemming's Row, St. Martin's Lane. A pure sample, consisting of pure tartaric acid, 1·5; sugar, water, and essential oil, 98·5.

No. 10.—From Camps', 47, South Audley Street. Similar to Nos. 7 and 8, consisting of tartaric acid, 1·9; sugar, water, and essential oil, 98·1.

The essential oil employed in all cases is that of lemon.

J. E. D. Rodgers.

*Laboratory, Grosvenor Place School of Medicine,*
*June* 21*st*, 1855.

The source of profit in the manufacture of the acidulated drops appears to consist in the use of small quantities of acid, and of as much water as will not prevent the formation of a solid drop. In moderate quantities, acid drops, of the kind referred to above, possess no injurious properties; on the contrary; they might be supplied to ships and expeditions, as an agreeable medium for the administration of vegetable acids for the prevention of scurvy.

ACIPENSER, the name of a genus of fishes of the chondropterygian or cartilaginous order. The genus contains the sturgeon, from which isinglass is produced; and the sterlet, which yields caviar. See *Caviar, Isinglass, Sterlet,* and *Sturgeon.*

ACORN, (Saxon *ac,* an oak; corn,) the fruit of the oak, or quercus, a tree of the natural order *Cupuliferæ* or *Amentaceæ,* (Linnean class and order, *Monœcia Polyandria.*) The acorn, according to the classic poets, formed the earliest food of man, its use having preceded that of corn: and it is considered as probable that the northern nations, among whom the oak is abundant, lived for a long time on this fruit. The Arcadians and early Spaniards regarded the acorn as a delicious article of food. Pliny states that the latter people roasted acorns in wood-ashes, and served them on their tables as dessert. In modern times acorns have been used in seasons of scarcity, as a substitute for corn. They are also used on the continent as a substitute for coffee, and as an adulteration of chicory in this country.

ADAM'S NEEDLE, the popular name of the yucca, an American plant of the natural order *Liliaceæ,* (Linnean class and order *Hexandria Monogynia*). The Indians make a kind of bread of the roots of this plant.

ADDER, (Saxon, *nœddre,*) a poisonous reptile of the vertebrate division, used formerly as a diet for invalids. See *Viper* and *Viper Broth.*

ADEPS, a technical term for fat or lard.

ADULTERATION, (*adulter,* mixed). In regard to foods and drinks, this term is used to imply the mixture with them of some substance or substances which do not enter necessarily into their composition. Taking the term in this extended sense it will be seen that food and drinks may be adulterated either *accidentally* or *intentionally*. The accidental adulterations arise mainly from three causes.

1. From the admixture of some substance used in the artificial preparation of the material, or entering into the composition of the apparatus used; as the admixture of sulphuric acid and salts of lead with citric acid.

2. From the accidental introduction or insufficient separation of foreign matter; as various organic matters in water; pieces of sugar-cane in sugar, &c.

3. From the presence of organised bodies or chemical products formed during a process of decomposition; as acari and fungi in sugar.

*Intentional* or *fraudulent* adulterations are very numerous. The principal objects with which they are perpetrated are:

1. To depreciate the real quality of a substance by adding to it some other substance, which may or may not be deleterious to the health. Examples of this form of adulteration are the addition of sand to sugar, of plaster of Paris to flour, potato-starch, bean-flour, and other meals to wheat-flour, &c.

2. To alter or heighten the physical properties of a substance. Thus, Prussian blue is said to be used by the Chinese to improve the appearance of the tea which is exported from their country; and an apparent strength is added to malt liquors by the use of *cocculus Indicus,* tobacco leaves, and the thorn-apple.

In regard to their effects on the body, adulterations may be either innocuous or deleterious. Under the latter division are principally found those of class 1, among the accidental adulterations; and of class 2, among the intentional.

In some cases it is difficult to draw the line of distinction; because, though the occasional use of a substance may not be injurious to health, its habitual use may be very hurtful.

The practice of wilful adulteration is probably of old date; but it has attracted a considerable amount of attention within the present century, both on account of the facilities which it has received from the advance of chemistry, and, on the other hand, on account of the readiness with which, by the aid of the above named science and of the microscope, the presence of articles used in the falsification of food can be detected. One of the earliest systematic works on the subject published in this country was that of Mr. Accum, which appeared about thirty years ago, the object of which was to point out the various culinary poisons. In 1848, a *Treatise on the Falsifications of Food* was published by Mr. John Mitchell, in which that author brought the subject well up to the advanced state of chemical knowledge. He does not appear, however, to have used the microscope in his investigations. In 1851, the *Lancet* commenced a series of searching inquiries into the purity of various ordinary articles of food. The work was intrusted to an "Analytical Sanitary Commission," who made numerous examinations, microscopical as well as chemical, of the several articles, and pointed out with uncompromising correctness, in many of them, an amount of adulteration which was scarcely imagined. Within the present year, Dr. Hassall, who conducted the investigations in the *Lancet,* has republished, in a separate volume, the articles from that journal.

In France, M. Chevallier published in 1854 a most elaborate work, entitled *Dictionnaire des Altérations et Falsifications des Substances Alimentaires, Médicamenteuses, et Commerciales,* (Dictionary of the Alterations and Falsifications of Alimentary, Medicinal, and Commercial Substances.) Information on the subject of adulteration is also afforded in Dr. Ure's *Dictionary of Science,* in Dr. Pereira's *Elements of Materia Medica,* and in Professor Johnston's *Chemistry of Common Life.*

(*To be continued.*)

